# Exploring the Effect of Driving Factors on Traffic Crash Risk among Intoxicated Drivers: A Case Study in Wujiang

**DOI:** 10.3390/ijerph16142540

**Published:** 2019-07-16

**Authors:** Zeyang Cheng, Zhenshan Zu, Jian Lu, Yunxuan Li

**Affiliations:** 1 Jiangsu Key Laboratory of Urban ITS, Southeast University, Nanjing 211189, China; 2 Jiangsu Province Collaborative Innovation Center of Modern Urban Traffic Technologies, Southeast University, Nanjing 211189, China; 3 School of Transportation, Southeast University, Nanjing 211189, China; 4 Traffic Police Brigade of Wujiang Public Security Bureau, Suzhou 215200, China

**Keywords:** intoxicated driving, traffic crashes, binary logistic regression, targeted policies

## Abstract

Intoxicated driving is a threat to both drivers and other road users. Exploring the association between intoxicated driving factors and traffic crashes is essential for taking effective countermeasures. Most previous works have studied the relation between intoxicated driving and traffic crash based on some large-sized cities. The current study aims to evaluate the effect of driving factors on traffic crashes among intoxicated drivers in a small-sized city in China. Descriptive statistics and binary logistic regression analysis are performed to guide the study, and the data (N = 1010) for the period 2016–2017 in Wujiang (i.e., a small-sized city in China) are employed as the target samples. The results demonstrate age, years of driving experience, road position, week, hour and blood alcohol concentration (BAC) are associated with traffic crashes in Wujiang. Specifically, the age of “18–25”, the years of driving experience of “≤2”, the “road intersection”, the “weekend”, the period of “0:00–6:59” and the BAC of “above 150 mg/100 mL” are more likely to cause traffic crashes among intoxicated drivers. The findings can be referred to make some targeted policies or measures to relieve Wujiang’s intoxicated driving situation and reduce the number of crashes caused by intoxicated driving.

## 1. Introduction

Traffic crashes under adverse driving environments such as intoxicated driving, speeding and other violation conditions cause a lot of social and economic losses in most countries [1,2]. In particular, alcohol-involved driving has been a significant road safety problem worldwide, as it is associated with visual impairment, a reduced reaction time, and impaired concentration, which increases crash risks, as well as their severity. The prevalence of alcohol among killed drivers in road crashes was approximately 25% in Norway, 31% in Finland, 32% in Sweden, 45% in Portugal [3], 40% in the U.S [4], and nearly 50% in Brazil [5]. In China, 5251 alcohol-related traffic crashes occurred in 2013, which caused 2228 deaths, 5291 serious injuries and 33,653,400 million RMB (5,384,533 million USD) economic loss; these figures represent increases of 6.90, 13.85 and 19.70%, respectively, compared with that of 2012 [6]. Thus, it can be seen that alcohol-related driving in China significantly increases the risk of casualty and economic loss, and also presents an upward trend. Therefore, alcohol-involved driving has become a social focus for legislative action. In China, the first comprehensive traffic law was passed in 2004 [7], and it defined drivers with a BAC between 20 mg/100 mL and 80 mg/100 mL as alcohol-involved offenders. These offenders will be punished with a license suspension of at least 6 months and a fine. The lesser offence of alcohol-involved driving is illegal but not a criminal case, so it is regulated by the civil legislation. A Chinese legislative amendment conducted on 1 May, 2011, defined intoxicated driving (i.e., BAC > 80 mg/100 mL) as a criminal case [8]. This amendment stipulates that an intoxicated driver is convicted of endangering public security with penalties including six-months imprisonment and a fine. Meanwhile, the driver’s license will be revoked, and the offenders cannot reapply for a license for five years. 

Generally, alcohol-involved driving can affect both motor vehicle drivers and pedestrians, and numerous literatures have alluded to this [3,4,9]. The prevalence of alcohol-involved driving was significantly associated with driver’s age and gender [10,11,12]. On the law enforcement aspect, Yannis et al. [13] proposed that law enforcement shows a better effect for relieving alcohol-involved driving under a scientific police practice. Some studies were proposed to evaluate the effect of legal punishment measures on alcohol-involved driving [14,15]. For example, license plate suspension was proven to be the most practical and effective measure against alcohol-involved driving, and the penalty point system ranks second, while a monetary fine appears to be inefficient [14]. Chang et al. [15] investigated the effectiveness of administrative lifetime license revocation (ALLR) on 768 alcohol-involved driving offenders in Taiwan, and the results showed that 23.4% offenders still drove almost the same as before, 59.8% drove significantly less frequently, and 16.8% complied completely with the ALLR restriction. On other aspects, Abayomi et al. [16] assessed the relationship between alcohol-involved driving and other risky behaviors by a stratified random sampling method. The results showed that alcohol-involved driving was associated with making phone calls, sending text messages and nonuse of helmets. 

The above studies have analyzed the contributors of alcohol-involved driving but fail to explore the association between alcohol-involved driving and traffic crashes. Stringer [17] explored the relationship between alcohol-involved driving and fatal crashes based on multilevel growth curve models. The findings indicated that the community norms, values, beliefs, and attitudes toward alcohol were related to fatal crashes. Tsui et al. [18] investigated the association between alcohol impairment and severe injury by a logistic regression model. The results illustrated that alcohol-involved driving crashes on weekdays cause a lower injury risk than those on weekends. Fujita and Shibata [19] clarified the relation between alcohol use and traffic fatalities in Japan. Their results showed that the traffic fatalities were significantly associated with road form under the alcohol consumption condition. Several scholars also evaluated the impact of BAC on traffic crashes [20,21,22], and their findings were almost similar (i.e., the crash risk increases as BAC increases).

Overall, prior studies have formed a broad research base for the relationship between alcohol-involved driving and traffic crashes, but these studies primarily concentrated on metropolises (i.e., [3,4,18,19,23,24]). However, very limited studies have focused on the small-sized cities of China. Compared with big-sized cities, small-sized cities are far behind in terms of city size, level of development, economic status, number of motor vehicles, demographic characteristics, education and law enforcement level [25]. These differences may cause a different alcohol-involved driving situation. Thus, the current study aims to analyze the influence factors of traffic crashes among intoxicated drivers (i.e., BAC >80 mg/100 mL) in a small-sized city in China, and to identify whether the influence factors are consistent with those of big-sized cities. Based on the results, countermeasures should be taken to prevent intoxicated driving-related crashes in Wujiang. If the influence factors are consistent with those of big-sized cities, the preventive measures or policies in previous studies can also be applied to Wujiang. Conversely, additional measures will be taken in Wujiang itself if the influence factors are different to those of big-sized cities.

## 2. Methods

### 2.1. Study Area and Data

**Figure 1:**
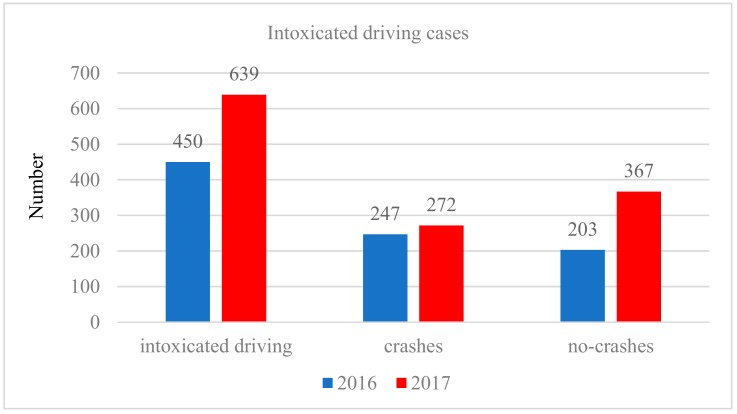
Intoxicated driving statistics of Wujiang for the years of 2016 and 2017.

Wujiang is administratively affiliated with Suzhou, China, and has a population of approximately 830,000 (the female population is approximately 410,000 and the male population is 430,000); the number of motor vehicles was more than 360,000, and the per capita GDP reached 73,106 RMB (11,623 USD) in 2017 [26]. An analysis of Wujiang may be representative among similar-level cities of China. First, the population, economic strength and vehicle numbers of Wujiang rank among the top ten in the same-sized cities of China, so alcohol-involved driving may be relatively prominent. Second, Wujiang has a high floating population. The large floating population may cover all levels of Chinese society, which makes the related factors such as occupation and education more representative. The damage caused by intoxicated driving is larger than that caused by common alcohol-involved driving, since it seriously affects drivers’ driving, as well as threatens the safety of other road users—that is why Chinese law regards this behavior as a criminal offence. Figure 1 depicts the statistics of intoxicated driving of Wujiang for the years of 2016 and 2017, and the traffic crash rates caused by intoxicated driving in 2016 and 2017 were 54.89 and 42.57%, respectively. 

The studied data were taken from the intoxicated driving crime database of Wujiang Public Security Bureau, and the database is maintained by the Traffic Police Brigade. The specific sample variables include gender, age, years of driving experience, occupation, education, vehicle nature, license plate, vehicle type, road type, road position, season, week, hour, public holiday, BAC, compulsory measure and crash information. Notably, all of the intoxicated samples are of two styles: one is crash cases (i.e., the crash reason is identified as drivers’ intoxicated driving by the traffic police, and the identification may be implemented by the targeted alcohol breathing test (i.e., BAC test) targeted at to the crash driver); another is the no-crash cases (i.e., intoxicated driving is identified by a random BAC test, and in this condition, the crash has not happened yet). After aggregation, these two types of data are integrated into the intoxicated driving database, among which, crash cases among intoxicated drivers were identified by a targeted BAC test, and the no-crash cases were identified by the random BAC test. The targeted BAC test targeted at the crash drivers may denote the intoxicated driving-related crashes situation in Wujiang. In respect to general drivers (those who did not cause a crash), the random BAC test targeted at them can be considered as the random sampling in the statistical analysis, which may represent the macro intoxicated driving situation in Wujiang. The above combinations of the two intoxicated driving samples can be used to explore the intoxicated driving factors and the crash factors of Wujiang. Then the raw samples include 1089 intoxicated driving cases (i.e., 639 cases in 2017, and 450 cases in 2016), but there were 1010 effective samples after dislodging the invalid information. 

Notably, traffic crash factors among intoxicated drivers are complex, as they may be influenced by many factors such as the weather, road infrastructure, and other uncertain conditions. In this study, we assumed that the other conditions are constant (i.e., we only considered the influence of factors involved in the intoxicated driving cases of Wujiang). Only in that case can this analysis, using Wujiang’s intoxicated driving samples, be meaningful. To determine the factors that are associated with traffic crashes, the dependent variable is set as follows: for the presence of crashes caused by intoxicated driving, “0” = no and “1” = yes. As the outcome measure is either 0 or 1, a binary logistic regression model was used to estimate the effect of the intoxicated driving-related factors on traffic crashes. 

In addition, it should be noticed that the samples in the random BAC test (i.e., the non-crash cases among intoxicated drivers) are insufficient, because a large-scale BAC test is impossible, and the samples used in this study (from 2016 to 2017) only represent some intoxicated driving samples, and this can be regarded as a random sampling in the statistic (Table 1). To solve this problem, we conducted an additional analysis with the non-alcohol-related crashes in Wujiang (from 2016 to 2017) and determined the influence factors of the non-alcohol-related crashes. Then we analyzed the results of the intoxicated driving-related crashes and non-alcohol-related crashes, which will make the results more convincing. The influence factors analysis results of the intoxicated driving-related crashes and non-alcohol-related crashes can be seen in Table 2 and Table 3.

### 2.2. Factors

The samples are classified as four groups: the driver factor (i.e., gender, age, years of driving experience, occupation and education), the vehicle factor (i.e., vehicle nature, license plate and vehicle type), the road factor (i.e., road type and road position) and other factors (i.e., season, week, hour, public holiday, BAC and compulsory measure). The specific classifications of these factors are as follows.

#### 2.2.1. Driver Factor

According to the typical driving habits and skills, ages in this study are classified into five levels (i.e., 18–25, 26–35, 36–45, 46–55, ≥56), then years of driving experience contains 7 levels: ≤2, 3–5, 6–10, 11–15, 16–20, ≥21, and others (e.g., unlicensed vehicle and license plate revoked), and this classification aligns with [23]. Occupation consists of unemployment, farmer, manual worker, general staff, civil servant, business, individual and other occupations. Then education is composed of illiteracy, primary school, junior middle school, high school, bachelor’s degree and higher, and other education. 

#### 2.2.2. Vehicle Factor

Vehicle nature includes operating vehicles and non-operating vehicles (i.e., private cars). Then license plates are divided into unlicensed vehicle, license plate suspended and normal license plate. Vehicle type contains four types: motorcycle, car, minibus and freight truck.

#### 2.2.3. Road Factor

Road type contains branch road, secondary road, main road, highway and others. This classification is similar to the standard partition of urban road in China. As for the road position, one is road intersection, and another is road section. There exist significant differences in quality and traffic characteristic between different road types and positions. So, having such a detailed classification of road type and position can provide a strong discrimination power in the intoxicated driving analysis.

#### 2.2.4. Other Factors

The format of intoxicated driving time in the database was “year–month–day–hour–minute–second”. For convenience, the intoxicated driving time is processed and converted into season, week, hour and public holiday, among which, season is categorized as spring (from March to May), summer (from June to August), autumn (from September to November) and winter (from December to February), which is in accordance with the division of solar terms in China. Week is divided into weekday (i.e., Monday, Tuesday, Wednesday, Thursday, and Friday) and weekend (Saturday and Sunday). Hour is classified into six groups: 00:00–06:59, 07:00–08:59, 09:00–11:59, 12:00–16:59, 17:00–19:59, and 20:00–23:59, which aligns with [23]. Public holidays refer to the holidays stipulated by the State Council of China, and they include New Year, Spring Festival, Qingming Festival, International Labor Day, Dragon Boat Festival, Mid-Autumn Festival and National Day. There are two labels in Public holidays: yes (i.e., it is a public holiday) and no (it is not a public holiday). 

**Figure 2:**
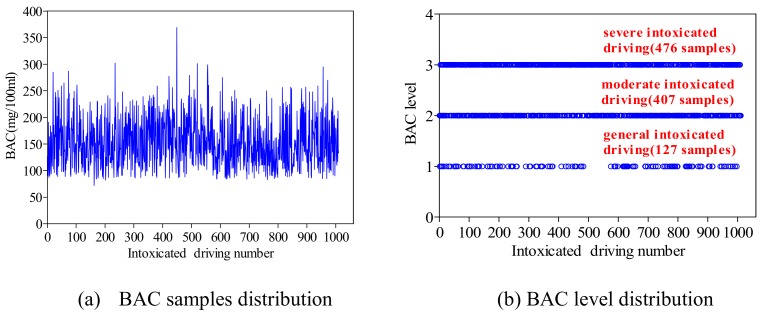
BAC and BAC level distribution among intoxicated drivers.

Because BAC in this study is all above 80 mg/100 mL (i.e., the intoxicated driving), it is classified as three levels in this study: 80 mg/100 mL–100 mg/100 mL (i.e., it is level 1, which is defined as the general intoxicated driving), 100 mg/100 mL–150 mg/100 mL (i.e., level 2, and it is defined as the moderate intoxicated driving), and above 150 mg/100 mL (i.e., level 3, and it is defined as the severe intoxicated driving). In the general intoxicated driving, moderate intoxicated driving and severe intoxicated driving groups are 127, 407, and 476 samples, respectively (see Figure 2). The compulsory measure is primarily detention—“yes” and “no” represent detention and no-detention, respectively. 

## 3. Analysis and Results

### 3.1. Descriptive Statistics

The independent variables (i.e., gender, age, years of driving experience, occupation, education, vehicle nature, license plate, vehicle type, road type, road position, season, week, hour, public holiday, BAC, compulsory measure) and the dependent variables (i.e., the crash) are presented in Table 1.

From Table 1, it is shown that the intoxicated driving offenders are more likely to be young (the proportion: “18–25” = 11.09%, “26–35” = 39.01%) and male (the proportion is 97.82%). Various occupations and education backgrounds are also associated with different intoxicated driving situations. For example, manual worker accounts for 37.62% of all occupations, and junior middle school accounts for 51.09% of all education backgrounds. For the vehicle factor, non-operating vehicles (99.11%), normal license plate (82.58%), and car (59.80%) show the largest percentages among the vehicle nature, license plate and vehicle type groups, respectively. As for the road factor, most of the intoxicated driving occurred on urban branch road (40.20%), followed by highway (31.77%). In addition, the percentages of road intersection and road section associated with intoxicated driving were 37.43 and 62.57%, respectively. As for the time aspect, the intoxicated driving cases that occurred in the evening (i.e., 20:00–23:59, the proportion is 47.52%) are more than those of other periods. Nevertheless, the intoxicated driving proportion is relatively balanced in seasonal distribution (the percentages of spring, summer, autumn and winter were 27.23, 24.16, 28.51 and 20.10%, respectively). In terms of week and public holiday, the weekday and non-public holiday obviously account for a larger proportion than weekend and public holiday. 

Table 1 also illustrates that severe intoxicated driving accounts for the largest proportion (47.13%), followed by moderate intoxicated driving (the proportion is 40.30%) and general intoxicated driving (the proportion is 12.57%). This reflects that most of the intoxicated driving cases in Wujiang are moderate and severe status (the total percentage is 87.43%). The crash rate caused by intoxicated driving is 46.93%, which is a rather high proportion. The above section presents the factor situation among the total intoxicated driving samples, but which factor has a significant impact on traffic crash has not been shown. Thus, the following sections will identify the related factors of traffic crashes in Wujiang. 

### 3.2. Binary Logistic Regression Analysis

To explore the factors and the extent to which they contribute to crashes under the intoxicated driving conditions, the binary logistic regression analysis is used to evaluate the effect of the intoxicated driving-related factors on traffic crashes (5% confidence level is set). The binary logistic regression model can be formulated as follows:

(1) $$\log it(\pi_{i})=\log[\frac{\pi_{i}}{1-\pi_{i}}]=\beta_{0}+\beta_{1}x_{1}+\ldots \beta_{j}x_{j}+\ldots \beta_{n}x_{n}$$

where $\pi_{i}$ is the probability of individual *I* leading to a crash after drinking; $x_{j}$j=1,2,…n are the influence factors (i.e., independent variables) such as age, years of driving experience, hour, etc.β is the coefficient of the independent variable, which determines the odds ratio (OR) of the independent variable. The OR is the relative amount by which the odds of the choice outcome increase or decrease when the value of the independent variable increases by one unit.

Before the binary logistic regression analysis, the independence test on the explanatory variables is performed, which illustrates that most of the explanatory variables are not correlated with each other. By the independence test, only the age and driving age show a very slight correlation (i.e., the correlation coefficient *R* = 0.159), which can be ignored for the convenience of the study. Then the correlation coefficients between other variables are all lower than 0.1. Then in the binary logistic regression analysis, the multiple classification variables need to be set as dummy variables, the contrast mode is selected as “indicator”, and the reference category is “first”. For example, gender (female), age (18–25), years of driving experience (≤2), etc., are the contrast bases; the other groups are compared based on these bases. The analysis results are shown in Table 2.

Age, years of driving experience, week, hour, road position and BAC are identified as the significant factors associated with traffic crashes. More specifically, compared with the offenders aged in the range 18–25, those aged in the range 36–45 are less likely to be involved in crashes (OR = 0.461), which suggests that the young intoxicated drivers may face a larger crash risk in Wujiang. Similarly, the novice driver (i.e., years of driving experience ≤2) will be more inclined to cause a traffic crash compared with the experienced drivers. Weekend (OR = 1.775, and 95% CI = 1.259–2.501) shows a higher crash risk than weekday. In regard of “hour”, traffic crashes among the periods of “12:00–16:59”, “17:00–19:59” and “20:00–23:59” are significant (*p*-value < 0.01), which illustrates that intoxicated drivers may face a crash risk in these periods. However, compared with the time of “0:00–6:59”, the above periods show a lower likelihood of traffic crash (OR < 1). This implies that “0:00–6:59” is the high occurrence period of traffic crash among intoxicated drivers in Wujiang. 

As for road position, “road section” (OR = 0.641, 95% CI = 0.483–0.850) demonstrates a lower association with traffic crashes than “road intersection”, so intoxicated drivers may present a higher crash risk at intersections than road sections. As for BAC, the group of “BAC > 150 mg/100 mL” shows a relatively high association (i.e., OR = 3.729, 95% CI = 2.395–5.806) with traffic crashes in Wujiang compared to other groups. In other words, the crash likelihood of “BAC = 100 mg/100 mL–150 mg/100 mL” and “BAC = 80 mg/100 mL–100 mg/100 mL” is less than that of “BAC > 150 mg/100 mL”. Then “BAC = 100 mg/100 mL–150 mg/100 mL” (OR = 1.069, 95% CI = 0.682–1.676) shows a relatively high crash risk compared to “BAC = 80 mg/100 mL–100 mg/100 mL”.

An additional comparison with non-alcohol-related crashes is made to further verify the above factors that influence the intoxicated driving-related crashes in Wujiang. This comparison aims to explore the factors associated with non-alcohol-related crashes, and to examine if these factors are consistent with that of the intoxicated driving-related crashes. With this in mind, the casualty crash data (i.e., non-alcohol-related crashes) of Wujiang from 2016 to 2017 is used to conduct the analysis. Notably, in the casualty crash dataset, occupation, education, public holiday, BAC, and compulsory measure are not included. Then the weather (i.e., 0, 1, 2, 3 represent the Sunny day, cloudy day, rainy day and snowy day, respectively), traffic crash type (i.e., 0, 1, 2, 3, 4, 5 denotes the unilateral crash, nonmotor vehicle–nonmotor vehicle crash, nonmotor vehicle–pedestrian crash, motor vehicle–nonmotor vehicle crash, motor vehicle–motor vehicle crash and motor vehicle–pedestrian crash, respectively) and crash casualties (i.e., casualties and no casualties are presented by 0 and 1, respectively.) are the new adding labels. The gender, age, years of driving experience, vehicle nature, license plate, vehicle type, season, hour, road position, and road type factors are consistent with those of the intoxicated driving dataset (see Table 1). Ignoring these small differences, the experimental results are unaffected. Finally, the analysis results by the binary logistic regression are shown in Table 3.

From Table 3, age (*p*-value = 0.039), vehicle type (*p*-value = 0.001), season (*p*-value = 0.013), and traffic crash type (*p*-value = 0.000) are the significant factors associated with the casualty traffic crash (i.e., non-alcohol-related crashes) of Wujiang. This is different to the influence factors of the intoxicated driving-related crashes (the significant factors include age, years of driving experience, week, hour, road position, and BAC). Only the age factor has a common influence on both the intoxicated driving-related crashes and non-alcohol-related crashes. As for other factors, the measures to prevent intoxicated driving-related crashes and casualty traffic crashes should be treated differently. For example, the target interventions focused on the prevention of the intoxicated driving-related crashes should consider the impact of age, years of driving experience, week, hour, road position, and BAC. However, age, vehicle type, season, and traffic crash type will be considered when making the corresponding policies to prevent traffic crash casualties. 

The above comparison further determined the factors (i.e., age, years of driving experience, week, hour, road position, and BAC) associated with the intoxicated driving-related crashes. The result could help target interventions focused on factors related to persons who drive while impaired, and it is also useful in providing enforcement with information that could help with distributing resources to prevent intoxicated driving. 

## 4. Discussion

In this study, we first analyze the factors related to the intoxicated driving of Wujiang, and found some results. In general, young and male drivers are more likely to drive intoxicated in Wujiang, which is consistent with [24,27,28,29,30], respectively. The major reason for the huge discrepancy between genders is the cardinality differences of drivers and drinkers between male and female drivers in China. In addition, female drivers are more cautious and less aggressive than male drivers in terms of driving attitude. Years of driving experience seems to show no obvious differences in the intoxicated driving cases of Wujiang, but it is certain that with more years of driving experience, intoxicated driving decreases to a certain extent (the years of driving experience proportion: “≤2” = 23.9%, “3–5” = 13.9%, “6–10” = 19.4%, “11–15” = 15.1%, “16–20” = 5.8% and “≥21” = 1.9%). This trend is different to the result of Zhang et al. (2014), as Zhang’s study indicated that among intoxicated drivers in Guangdong (the large-sized city), the years of driving experience proportion of “≤2” is lower than “3–5”, “6–10” and “16–20”. This may be attributed to the complex road conditions, and huge number of motor vehicles in Guangdong. In this situation, novice drivers drive carefully and they do not drink before they drive, especially during the peak hour. In contrast, the situation in Wujiang is relatively relaxed, and novice drivers need not be worried about the traffic condition, because the traffic operation status of Wujiang is smooth throughout the year [31]. The results also demonstrate that the manual worker exhibits higher intoxicated driving behavior than other occupations. Since manual workers are under pressure in their professional and social lives, a negative mood may manifest itself in drinking. Concurrently, because of their low awareness of traffic laws, their intoxicated driving behaviors are frequent. In addition, the offenders with junior middle school education comprise the biggest percentage among the education backgrounds, followed by primary school education, high school education, and bachelor’s degree and higher education. Overall, the intoxicated driving situation decreases with the advancement of drivers’ education backgrounds. The different intoxicated driving situations among various occupations and levels of education indicate that drivers with poor education and low income are more inclined to drive intoxicated because of their low awareness of traffic law. A high prevalence of intoxicated driving is presented at branch road and highway (especially the rural highway)—the total proportion of these two road types is 71.97%. This can be ascribed to the fact that the law enforcement in these areas has increased in recent years in Wujiang, because the traffic safety of urban branch road and rural areas has attracted attention [31]. Thus, in this situation, intoxicated drivers may more frequently encounter a BAC test on other road types. The intoxicated driving samples in the road section are larger than those of the road intersection—this may be attributed to the fact that the BAC tests are performed more in the road section than the intersection in China. As BAC tests at intersections have a huge impact on traffic operation, especially during the peak hours, the vehicles need to stop nearby the intersection when they are called to complete the BAC test, which will block the traffic upstream of the intersection. By contrast, the BAC tests in the road section have a relatively small effect on traffic operation. In addition, intoxicated driving in Wujiang is more likely to take place during the evening and at dawn (i.e., 20:00–23:59 and 00:00–06:59), which aligns with [5,23]. 

In addition, some factors related to traffic crashes among intoxicated drivers in Wujiang have also be identified. Overall, the influence factors related to traffic crashes are age (*p*-value = 0.013), years of driving experience (*p*-value = 0.021), week (*p*-value = 0.001), hour (*p*-value = 0.004), road position (*p*-value = 0.002) and BAC (*p*-value = 0.000). Specifically, the risk of crash occurring among young intoxicated drivers (aged in the range 18–25) is larger than that of middle-aged intoxicated drivers (i.e., aged in the range 36–45), which aligns with [32,33]. This difference may be explained by two aspects: one is that young drivers may show low drinking capacity and driving experience compared with middle-aged drivers, so their crash hazard handling capacity is weaker than that of the middle-aged groups; on the other hand, young drivers are more inclined to drive dangerously than middle-aged groups, which may put them into a more dangerous situation, and this view is also supported by [24,34]. Novice drivers are more likely to cause a crash in the intoxicated driving environment in contrast to experienced drivers (OR < 1), which is in accordance with [33]. This can be attributed to the weak crash hazard avoidance capacity of novice drivers. Weekend (OR = 1.775, 95% CI = 1.259–2.501) shows a bigger crash risk than weekday, which is consistent with the consumption of alcohol at bars and parties in Wujiang. This result is in line with [5,18]. Because people have more time to attend parties and recreational activities at weekends, the chances of drinking are greatly increased. This situation is consistent with [24]. Among the hour groups, the period 0:00–6:59 shows the highest crash risk—the same trend as [5]. The crash risk under the high BAC conditions is high (i.e., the crash likelihood of “BAC > 150 mg/100 mL” is the biggest, followed by “BAC = 100 mg/100 mL–150 mg/100 mL” and “BAC = 80 mg/100 mL–100 mg/100 mL”), which also aligns with previous studies about large cities. 

The only difference to that of large cities may be the road position analyses. This is because, in previous studies, no obvious association has been found between the road position (including the road intersection and road section) factors and traffic crashes among intoxicated drivers. However, in this study, the road position is associated with traffic crashes among intoxicated drivers in Wujiang. Specifically, the intersection presents a greater association with crashes than the road section (OR = 0.641) among Wujiang’s intoxicated drivers, and this may be determined by the complicated traffic conditions of the road intersection. For example, at the road intersection, the conflict points increase, the “vehicles stop and go” phenomenon exists when approaching the intersections, and some traffic violations such as red-light running and malicious lane-changing are prominent. All the above increases the crash risk at intersections. In the intoxicated driving condition, the above phenomenon is more serious, which further raises the crash risk. By contrast, the situation at road sections is relatively steady, especially in small cities. In small cities, the traffic flow in road sections is low, and the traffic operation is good as a whole. In this case, the interference between vehicles is weak. 

## 5. Conclusions

This study employs a dataset from Wujiang to evaluate the association between driving factors and traffic crashes among intoxicated drivers. The results reveal that some factors (i.e., age, years of driving experience, road position, week, hour and BAC) are significantly associated with traffic crashes in Wujiang among intoxicated drivers, especially those in the age range of “18–25”, with years of driving experience of “≤2”, the “road intersection”, the “weekend”, the period of “0:00–6:59” and BAC above 150 mg/100 mL. Among these factors, age, years of driving experience, week, hour and BAC are also identified in previous studies. However, road position merits special attention as the analysis result is different to previous studies on large cities. Specifically, the intersection presents a larger crash likelihood among intoxicated drivers in Wujiang. Therefore, the targeting policies and measures to tackle intoxicated driving related to crashes should pay more attention to drivers at the road intersection when preventing traffic crashes. For example, regarding traffic law enforcement, the law enforcement officers need to focus on intoxicated driving at intersections, especially those key intersections of Wujiang. When combining the overlap effect of the time factor, age factor, week factor, road position factor, etc., the measures can be more effective at reducing the number of traffic crashes caused by intoxicated driving (for example, the BAC test may be more targeted, and it should be carried out more during the period of “0:00–6:59” at the weekend, and mainly complete by young drivers at intersections). Naturally, Wujiang should also take the common measures that align with previous studies on large cities. 

Overall, the measures to prevent intoxicated driving and its related crashes should consider the effect of both the special factors (i.e., the road intersection) that are different to large cities and the regular factors (i.e., the age of “18–25”, the years of driving experience of “≤2”, the “weekend”, the period of “0:00–6:59”, etc.) that align with large cities. Only in this case can the governance of Wujiang’s intoxicated driving be more effective. Finally, the findings of the Wujiang study may be representative, and may likely be generalized to other small-sized cities (especially those that show a similar level to Wujiang) of China. In particular, since we consider the problems with respect to intoxicated driving in Wujiang, which is typical in reflecting the issues faced by other small-sized cities of China, our findings can serve as a reference for providing the preventative measures and policies of this type of city. 

In conclusion, intoxicated driving-related crashes have become an important cause of fatalities and injuries in Chinese small-sized cities in recent years. By examining the factors that determine the association between intoxicated driving and traffic crashes, the corresponding measures can be taken, which will greatly reduce the number of traffic crash losses. 

## 6. Limitation

Several limitations of this study should be stated because of the constraints of the current experiment’s condition and data acquisition. First, some influence factors such as road alignment, pavement humidity and other undetermined factors, are not provided by the database of Wujiang, so the evaluation results of Wujiang may tend to be macroscopic. In addition, the studied data of the intoxicated driving samples may represent only some intoxicated drivers in Wujiang because of the random BAC test targeted at the drivers. Some intoxicated driving may not be caught, because not all the time periods and locations included a BAC test; after all, the police resources are limited. Under this condition, the factors (e.g., the traffic control, a more exhaustive BAC testing and other traffic data) that may affect the analysis results are neglected because of the limitations of data acquisition. The above issues still exist objectively, which may be difficult to solve under the current experiment conditions. However, future studies will be conducted, will collect a more comprehensive dataset and consider the undetermined factors in this study, and in that case, the above issues will be solved. 

## Figures and Tables

**Table 1:** Descriptive statistics of the variables (N = 1010).

Factor	N (%)	Factor	N (%)
**Gender**		Motorcycle (0)	214 (21.19%)
Female (0)	22 (2.18%)	Car (1)	604 (59.80%)
Male (1)	988 (97.82%)	Minibus (2)	182 (18.02%)
**Age**		Freight truck (3)	10 (0.99%)
18–25 (0)	112 (11.09%)	**Road type**	
26–35 (1)	394 (39.01%)	Branch road (0)	406 (40.20%)
36–45 (2)	295 (29.21%)	Secondary road (1)	13 (1.29%)
46–55 (3)	183 (18.12%)	Main road (2)	257 (25.45%)
≥56 (4)	26 (2.57%)	Highway (3)	321 (31.77%)
**Years of driving experience (year)**		Other roads (4)	13 (1.29%)
≤2 (0)	131 (12.97%)	**Road position**	
3–5 (1)	143 (14.16%)	Road intersection (0)	378 (37.43%)
6–10 (2)	185 (18.32%)	Road section (1)	632 (62.57%)
11–15 (3)	147 (14.55%)	**Season**	
16–20 (4)	49 (4.85%)	Spring (0)	275 (27.23%)
≥21 (5)	18 (1.78%)	Summer (1)	244 (24.16%)
Others (6)	337 (33.37%)	Autumn (2)	288 (28.51%)
**Occupation**		Winter (3)	203 (20.10%)
Unemployment (0)	69 (6.83%)	**Week**	
Farmer (1)	9 (0.89%)	Weekday (0)	808 (80%)
Manual worker (2)	380 (37.62%)	Weekend (1)	202 (20%)
General staff (3)	169 (16.73%)	**Hour**	
Civil servant (4)	15 (1.49%)	0:00–6:59 (0)	248 (24.55%)
Businessman (5)	46 (4.55%)	7:00–8:59 (1)	9 (0.89%)
Individual (6)	239 (23.66%)	9:00–11:59 (2)	11 (1.10%)
Others (7)	83 (8.22%)	12:00–16:59 (3)	83 (8.22%)
**Education**		17:00–19:59 (4)	179 (17.72%)
Illiteracy (0)	18 (1.78%)	20:00–23:59 (5)	480 (47.52%)
Primary school (1)	177 (17.52%)	**Public holiday**	
Junior middle school (2)	516 (51.09%)	No (0)	975 (96.53%)
High school (3)	172 (17.03%)	Yes (1)	35 (3.47%)
Bachelor’s and higher (4)	118 (11.69%)	**BAC (mg/100 mL)**	
Others (5)	9 (0.89%)	80–100 (0)	127 (12.57%)
**Vehicle nature**		100–150 (1)	407 (40.30%)
Operating vehicles (0)	9 (0.89%)	>150 (2)	476 (47.13%)
Non-operating vehicles (1)	1001 (99.11%)	**Compulsory measure**	
**License plate**		No (0)	986 (97.62%)
Unlicensed vehicle (0)	119 (11.78%)	Yes (1)	24 (2.38%)
License plate suspended (1)	57 (56.44%)	**Crash**	
Normal license plate (2)	834 (82.58%)	No (0)	536(53.07%)
**Vehicle type**		Yes (1)	474(46.93%)

**Table 2:** Significant factors associated with intoxicated driving-related crashes in Wujiang.

Factor	df	*p*-Value	OR	95.0% CI for OR	
				Lower	Upper
Age (base: age (18–25))	4	0.013 ^*^			
Age (26–35)	1	0.282	0.769	0.476	1.242
Age (36–45)	1	0.003 ^**^	0.461	0.274	0.773
Age (46–55)	1	0.331	0.752	0.424	1.335
Age (≥56)	1	0.368	0.620	0.219	1.754
Years of driving experience (base: ≤2)	6	0.021 ^*^			
Years of driving experience (3–5)	1	0.011 ^*^	0.500	0.293	0.854
Years of driving experience (6–10)	1	0.022 ^*^	0.549	0.328	0.918
Years of driving experience (11–15)	1	0.005 ^**^	0.446	0.254	0.783
Years of driving experience (16–20)	1	0.008 ^**^	0.345	0.156	0.760
Years of driving experience (≥21)	1	0.005 ^**^	0.135	0.034	0.542
Years of driving experience (others)	1	0.008 ^**^	0.533	0.336	0.846
Week (weekend)	1	0.001 ^**^	1.775	1.259	2.501
Hour (base: hour (0:00–6:59))	5	0.004 ^**^			
Hour (7:00–8:59)	1	0.160	0.341	0.076	1.530
Hour (9:00–11:59)	1	0.244	0.443	0.113	1.743
Hour (12:00–16:59)	1	0.004 ^**^	0.410	0.225	0.748
Hour (17:00–19:59)	1	0.000 ^**^	0.454	0.292	0.705
Hour (20:00–23:59)	1	0.001 ^**^	0.566	0.399	0.803
Road position (Road section)	1	0.002 ^**^	0.641	0.483	0.850
BAC (base: BAC (80–100))	2	0.000 ^**^			
BAC (100–150)	1	0.772	1.069	0.682	1.676
BAC (>150)	1	0.000 ^**^	3.729	2.395	5.806
Constant	1	0.012	2.445		

^*^p < 0.05; ^**^ *p* < 0.01.

**Table 3:** Significant factors associated with non-alcohol-related crashes in Wujiang.

Factor	df	*p*-Value	OR	95.0% CI for OR	
				Lower	Upper
Age (base: age (18–25))	4	0.039 ^*^			
Age (26–35)	1	0.081	0.218	0.039	1.204
Age (36–45)	1	0.192	0.314	0.055	1.792
Age (46–55)	1	0.008 ^**^	0.092	0.016	0.538
Age (≥56)	1	0.728	0.769	0.175	3.382
Vehicle type (base: motorcycle)	2	0.001 ^**^			
Vehicle type (car)	1	0.001 ^**^	0.061	0.012	0.306
Vehicle type (minibus)	1	0.000 ^**^	0.037	0.006	0.219
Season (base: spring)	3	0.013 ^*^			
Season (summer)	1	0.235	0.522	0.179	1.527
Season (autumn)	1	0.004 ^**^	0.148	0.041	0.536
Season (winter)	1	0.012 ^*^	0.180	0.047	0.689
Traffic crash type (base: unilateral crash)	5	0.000 ^**^			
Traffic crash type (non-motor vehicles–non-motor vehicles)	1	0.999	0.000	0.000	-
Traffic crash type (non-motor vehicles–pedestrians)	1	0.008 ^**^	0.005	0.000	0.248
Traffic crash type (motor vehicles–non-motor vehicles)	1	0.000 ^**^	0.030	0.004	0.213
Traffic crash type (motor vehicles–motor vehicles)	1	0.200	0.315	0.054	1.842
Traffic crash type (motor vehicles–pedestrians)	1	0.000 ^**^	0.020	0.003	1.156
Constant	1	0.007	3.879		

^*^p < 0.05; ^**^ *p* < 0.01.
